# Impact of oral osteoarthritis therapy usage among other risk factors on knee replacement: a nested case-control study using the Osteoarthritis Initiative cohort

**DOI:** 10.1186/s13075-018-1656-2

**Published:** 2018-08-07

**Authors:** Marc Dorais, Johanne Martel-Pelletier, Jean-Pierre Raynauld, Philippe Delorme, Jean-Pierre Pelletier

**Affiliations:** 1 StatSciences Inc., Notre-Dame-de-l’Île-Perrot, Quebec, Canada; 2 Osteoarthritis Research Unit, University of Montreal Hospital Research Centre (CRCHUM), Montreal, Quebec Canada

**Keywords:** Nested case-control, Osteoarthritis, Osteoarthritis Initiative, Knee replacement, Acetaminophen, NSAIDs, Coxibs, Narcotics, Glucosamine/chondroitin sulfate

## Abstract

**Background:**

The aim of this study was to measure the association between exposure to commonly used oral osteoarthritis (OA) therapies and relevant confounding risk factors on the occurrence of knee replacement (KR), using the Osteoarthritis Initiative (OAI) database.

**Methods:**

In this nested case-control design study, participants who had a KR after cohort entry were defined as “cases” and were matched with up to four controls for age, gender, income, Western Ontario and McMaster Universities Osteoarthritis Index (WOMAC) pain, Kellgren-Lawrence grade, and duration of follow up. Exposure to oral OA therapies (acetaminophen, non-steroidal anti-inflammatory drugs (NSAIDs), cyclooxygenase-2 (COX-2) inhibitors, narcotics, and glucosamine/chondroitin sulfate) was determined within the 3 years prior to the date of the KR. Conditional regression analyses were performed to estimate the association between KR and exposure to oral OA therapies and other potential confounding risk factors.

**Results:**

A total of 218 participants who underwent a KR (cases) were matched to 540 controls. The median time to KR was 4.3 years among cases. The majority in both groups were Caucasian with mean age of 69 years and 61% were female. Numerically, cases were more exposed to acetaminophen, NSAIDs, and COX-2 inhibitors. Exposure to narcotics and glucosamine/chondroitin sulfate was relatively similar between cases and controls. No significant association was found between the occurrence of KR and exposure to any of the oral OA therapies within the 3 years prior to KR. A significantly higher occurrence of KR was found in Caucasian subjects (OR 1.84; 95% CI, 1.13–2.99; *p* = 0.015) and subjects with body mass index (BMI) ≥ 27 kg/m^2^ (OR 1.65; 95% CI, 1.06–2.58; *p* = 0.027).

**Conclusion:**

This study provides evidence that the main risk factors leading to KR are disease severity, symptoms and high BMI. Importantly, exposure to oral OA therapies was not associated with the occurrence of KR.

## Background

Osteoarthritis (OA) is one of the arthritis conditions most often associated with chronic pain and disability [1]. As such, patients with OA are in need of treatment that includes a number of different pharmacological classes of agents. They are most commonly oral agents and are very often prescribed for chronic administration over an extended period of time. In recent years there has been concern about the safety of some of drug treatments, mainly related to potential detrimental systemic effects such as cardiovascular risks and morbidity associated, for instance, with non-steroidal anti-inflammatory drugs (NSAIDs) and coxibs [2]. Moreover, the use of narcotics by patients with OA has also been associated with an increased risk of morbidity and even mortality [3]. Concerns have also been raised about the effects of these drug treatments on the evolution of OA structural changes, particularly in weight-bearing joints such as the hip and knee [4–11]. The impact of such oral treatments, especially NSAIDs, on OA disease progression and outcome, whether negative or positive, remains at this time an open question that needs to be further explored.

Studying the effects of different therapeutic classes of drugs used for the treatment of OA and their potential impact on disease progression is not an easy task. However, the use of observational cohorts provides a real-life scenario [8, 10, 11]. The Osteoarthritis Initiative (OAI) cohort presents several advantages for such purpose owing to its size, duration, and very large amount of comprehensive demographic and clinical information available on the study participants, including drug treatment. Structural changes are also assessed by imaging using knee x-rays and magnetic resonance imaging (MRI). The latter has proven to be extremely reliable, sensitive, and very useful for studying disease outcomes [10–15]. Joint replacement is considered a clinically relevant disease outcome in knee OA, which is related to both disease symptoms and structural damage [14–18]. Using the OAI cohort, the objective of this nested case-control study was to explore the potential effects of the most commonly used drug treatments for knee OA, while controlling for the most relevant confounding risk factors on the occurrence of knee replacement (KR). One of the main reasons for choosing a nested case-control design was that, in addition to the robustness of this approach, the exposure to oral OA therapies could be measured in different time windows before the KR.

## Methods

### Study population

Participants were selected from the OAI database, which is publicly available at https://oai.epi-ucsf.org/datarelease/. The OAI established and maintains a natural history database for knee OA that includes clinical evaluation data and radiological and magnetic resonance (MR) images of 4796 (including the controls) men and women aged 45–79 years at the time of enrolment (cohort entry) between February 2004 and May 2006. The participants selected for the study were from both the progression and the incidence subcohorts. In brief, the participants in the progression subcohort (*n* = 1389) were subjects with symptomatic tibiofemoral knee OA at baseline who had both of the following in at least one knee at baseline: frequent knee symptoms in the past 12 months defined as “pain, aching or stiffness in or around the knee on most days” for at least 1 month during the past 12 months, and radiographic tibiofemoral knee OA, defined as definite tibiofemoral osteophytes (Osteoarthritis Research Society International (OARSI) atlas grades 1–3), equivalent to Kellgren-Lawrence (KL) grade ≥ 2 on the fixed-flexion radiograph. The participants in the incidence subcohort (*n* = 3285) did not have symptomatic knee OA, as defined above, in either knee at baseline. However, they had characteristics that placed them at increased risk of developing symptomatic knee OA during the study. The specific eligibility risk factor criteria for the incidence subcohort were: knee symptoms in a native knee in the past 12 months; being overweight defined using gender and age-specific cut-off points for weight; knee injury defined as a history of knee injury causing difficulty walking for at least a week; knee surgery including meniscal and ligamentous repairs and unilateral total KR for OA; family history defined as a knee replacement for OA in a biological parent or sibling; Heberden’s nodes; repetitive knee bending at work or outside work; and age 70 to 79 years.

In this nested case-control study, participants are defined as subjects presenting a first occurrence of a KR procedure between February 2004 and October 2015. The date on which the KR was reported is defined as the index date. For each case, up to four control subjects [19] without a history of KR before the index date were matched for age (± 1 year), gender, and index date information on income level (± 40,000USD), Western Ontario and McMaster Universities Osteoarthritis Index (WOMAC) pain (± 10%), KL grade (same grade), and duration of follow up. Subjects who had missing information on KR or matching variables were excluded.

### Definition of exposure to oral OA therapies

The oral OA therapies included acetaminophen, NSAIDs, cyclooxygenase-2 (COX-2) inhibitors, narcotics, and glucosamine/chondroitin sulfate. The information concerning the use of these therapies was obtained from the medical history in the OAI database (typical question: “During the past 6 months (or 30 days), did you use *(specific therapy)* for joint pain or arthritis on most days?”) For the primary objective, exposure to each of these classes of oral OA therapies was measured within the 3 years preceding the index date. Exposure was defined as the percentage (%) of all available follow ups at which the subjects reported currently using, at the time of the query, oral OA therapies. Hence, categories of exposure were defined as “no exposure”, “exposure of 1–79%”, and “exposure of 80% or more”. For the secondary objectives, different time periods were employed to measure the exposure to oral OA therapies: 2 years, 4 years, and 5 years before the index date. Subjects who had missing information on exposure to oral OA therapies were excluded.

### Covariates

The covariates were race, education level, body mass index (BMI), WOMAC scores (stiffness, function, total), knee injury and osteoarthritis outcome scores (KOOS) (pain, symptoms, and quality of life (QoL)), joint space width (JSW), cartilage volume, bone marrow lesions (BML) size, and presence of meniscal extrusion. The WOMAC [20] and KOOS [21] questionnaires are self-administered: higher WOMAC scores and lower KOOS scores indicate more pain/symptoms and greater functional impairment. Covariates were measured at the index date or at the last available visit before the index date.

### Clinical and demographics data

The clinical data were extracted from the OAI database. These included variables used for the matching (age, gender, income level, WOMAC pain, KL), covariates, and arthritis drug treatment taken by the patients, which included acetaminophen, NSAIDs, COX-2 inhibitors, narcotics, and glucosamine/chondroitin sulfate.

### Imaging characteristics

The KL grade and the JSW data were obtained from the OAI database (central reading). The MR images were acquired from 3.0 T apparatus (Magnetom Trio, Siemens) at the four OAI clinical centers using a double-echo steady-state imaging protocol. Fully automated and validated quantitative MRI technology was used to assess the cartilage volume [12, 22] and the BMLs [23], and a validated scoring method for the meniscal extrusion [24].

 Cartilage volume was analyzed in the knee (femur and plateau) and in the medial and lateral compartments. The change over time was assessed as previously described [12]. Quantitative BML assessment was expressed as a percentage (%) of the lesion in the bone volume in each region of interest [23]. Meniscal extrusion was scored as absence or presence of partial or complete extrusion detected in any of the three segments of the meniscus [24, 25].

### Statistical analyses

Descriptive analyses of sociodemographic and clinical characteristics were conducted for case and control patients. These included matching variables (gender and, at index date, age, income level, WOMAC pain, and KL grade), the aforementioned covariates, and exposure to the different classes of oral OA therapies in the 3 years prior to KR. Proportions were calculated for categorical variables, and median and interquartile range (IQR) for continuous variables.

The association between the occurrence of KR and sociodemographic/clinical characteristics (not those used in the matching between cases and controls) was measured using crude conditional logistic regression. An adjusted regression model including significant covariates and pertinent clinical variables was employed to determine the association between exposure to oral OA therapies and occurrence of KR. Odds ratios (OR) and 95% confidence interval (CI) were calculated. Only data with sufficient patient number (*n* > 10) per time exposure were analyzed and presented. A two-tailed *p* value <0.05 was considered significant. All statistical analyses were performed using SAS software, V.9.3 (SAS Institute, Cary, NC, USA).

## Results

**Fig. 1:**
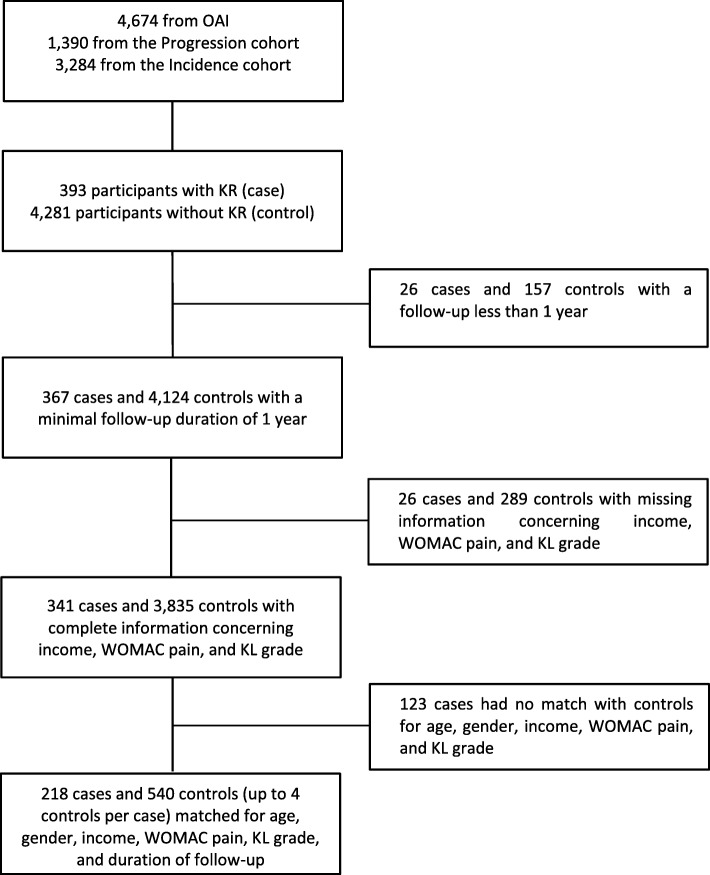
Selection of knee replacement cases and controls. KL, Kellgren-Lawrence; KR, knee replacement; OAI, Osteoarthritis Initiative; WOMAC, Western Ontario and McMaster Universities Osteoarthritis Index

Of the 4674 participants from the incidence and progression subcohorts enrolled in the OAI, 393 had a KR during the follow up. After exclusions for follow up less than 1 year, missing information for matching variables, or no possible match with at least one control, a total of 218 cases were matched to 540 controls for age, gender, income level, WOMAC pain, KL grade, and duration of follow up (Fig. 1).

### Characteristics at index date

For the cases (Table 1) the mean age was 68.9 years, 60.6% were female, and the median (IQR) time from cohort entry to having a KR was 4.3 years (1.0–8.9). The majority of cases and controls were white/Caucasian, had an income level greater than $50,000, and had either some college education or a graduate degree. The proportion of cases with a BMI of 27 kg/m^2^ or higher was 75% compared to 71% in controls (Table 2). Compared to controls, cases had higher WOMAC scores (except stiffness), smaller joint space width (JSW), and globally had more BMLs in the knee. Cases and controls had similar characteristics in terms of cartilage volume and meniscal extrusion.

**Table 1:** Sociodemographics at index date

	Knee replacement	
	Case (*n* = 218)	Control (*n* = 540)
Time to KR after cohort entry, % (*n*)		
Between year 1 and year 2	10.6% (23)	–
Between year 2 and year 3	15.1% (33)	–
Between year 3 and year 4	18.8% (41)	–
Between year 4 and year 5	17.9% (39)	–
Between year 5 and year 6	12.8% (28)	–
Between year 6 and year 7	7.8% (17)	–
Between year 7 and year 8	8.3% (18)	–
Between year 8 and year 9	8.7% (19)	–
Median (IQR) (years)	4.3 (1.0–8.9)	
OAI subcohort, % (*n*)		
Progression	61.9% (135)	58.5% (316)
Incidence	38.1% (83)	41.5% (224)
Age (years), median (IQR)	68.9 (61.8–74.3)	68.6 (61.6–73.8)
Female, % (*n*)	60.6% (132)	61.1% (330)
Race, % (*n*)	*(n = 217)*	
White or Caucasian	83.9% (182)	78.0% (421)
Black or African American	12.5% (27)	19.4% (105)
Asian	1.8% (4)	0.4% (2)
Other non-white	1.8% (4)	2.2% (12)
Income level USD, % (*n*)		
Less than $25,000	10.6% (23)	12.4% (67)
$25,000 to < $50,000	28.0% (61)	30.6% (165)
$50,000 to < $100,000	38.0% (83)	38.9% (210)
$100,000 or greater	23.4% (51)	18.1% (98)
Education level, % (*n*)		
Less than high school graduate	0.9% (2)	4.1% (22)
High school graduate	19.3% (42)	10.9% (59)
Some college	26.6% (58)	30.7% (166)
College graduate	18.3% (40)	18.2% (98)
Some graduate school	6.9% (15)	7.2% (39)
Graduate degree	28.0% (61)	28.9% (156)

Data shown are proportion of patients (%), number of patients (*n*), age in years, or median (interquartile range (IQR))

*KR* knee replacement, *OAI* Osteoarthritis Initiative

**Table 2:** Clinical characteristics at index date

	Knee replacement	
	Case (*n* = 218)	Control (*n* = 540)
	*(n = 204)*	*(n = 537)*
BMI ≥ 27 kg/m^2^, % (*n*)	75.0% (153)	70.6% (379)
WOMAC, median (IQR)		
Pain (0–20)	6.0 (4.0–9.0)	5.0 (3.0–8.0)
Function (0–68)	22.2 (14.9–31.0)	18.0 (8.5–25.5)
Stiffness (0–10)	3.0 (2.0–4.0)	3.0 (2.0–4.0)
Total (0–98)	31.2 (21.8–43.0)	25.0 (13.0–37.0)
KOOS, median (IQR)		
Pain (0–100)	61.1 (47.2–72.2)	66.7 (55.6–79.3)
Symptoms (0–100)	64.3 (50.0–78.6)	75.0 (58.9–85.7)
QoL (0–100)	43.8 (31.3–56.3)	56.3 (43.8–68.8)
Kellgren-Lawrence grade, % (*n*)		
0	1.4% (3)	1.7% (9)
1	3.7% (8)	3.7% (20)
2	19.3% (42)	23.9% (129)
3	31.6% (69)	29.8% (161)
4	44.0% (96)	40.9% (221)
	*(n = 211)*	*(n = 524)*
JSW (mm), median (IQR)	2.0 (0.8–4.6)	2.8 (1.1–4.6)
Cartilage volume (mm^3^), median (IQR)	*(n = 201)*	*(n = 510)*
Global knee	11,037 (9127 - 13,634)	11,058 (9351 - 13,508)
Medial compartment	5127 (3971 - 6482)	5219 (4176 - 6502)
Lateral compartment	6028 (4864 - 7480)	6043 (4970 - 7265)
BML	*(n = 201)*	*(n = 511)*
Global		
Median (IQR)	2.0 (0.6–4.4)	1.7 (0.4–3.8)
BML ≥ 1%, % (*n*)	65.7% (132)	61.5% (314)
Medial compartment		
Median (IQR)	0.014 (0.0–0.058)	0.021 (0.0–0.057)
BML ≥ 1%, % (*n*)	34.9% (76)	42.2% (228)
Lateral compartment		
Median (IQR)	0.005 (0.0–0.025)	0.0 (0.0–0.018)
BML ≥ 1%, % (*n*)	23.9% (52)	20.7% (112)
	*(n = 215)*	*(n = 532)*
Meniscal extrusion, % (*n*)	39.5% (85)	38.5% (205)

Data shown are proportion of patients (%), number of patients (n), or median (interquartile range (IQR))

*BMI* body mass index, *BML* bone marrow lesions, *JSW* joint space width, *KOOS* Knee injury and Osteoarthritis Outcome Score, *QoL* quality of life, *WOMAC* Western Ontario and McMaster Universities Osteoarthritis Index

Exposure to narcotics and glucosamine/chondroitin sulfate treatment in the 3 years prior to index date was similar between cases and controls (Table 3). Cases were, however, numerically more exposed to acetaminophen, NSAIDs, and COX-2 inhibitors. Due to absence of specific NSAID description in the OAI database, it was not possible to analyze the effects of the different NSAIDs separately.

**Table 3:** Exposure to different oral OA therapies in the 3 years prior to index date

	Knee replacement	
Oral OA therapies, % (*n*)	Case (*n* = 161)^a^	Control (*n* = 360)^a^
Acetaminophen		
No exposure	70.2% (113)	75.0% (270)
Exposure 1–79%	26.7% (43)	22.5% (81)
Exposure ≥ 80%	3.1% (5)	2.5% (9)
NSAIDs		
No exposure	37.3% (60)	51.4% (185)
Exposure 1–79%	38.5% (62)	33.3% (120)
Exposure ≥ 80%	24.2% (39)	15.3% (55)
COX-2 inhibitors		
No exposure	86.3% (139)	91.1% (328)
Exposure 1–79%	7.5% (12)	4.7% (17)
Exposure ≥ 80%	6.2% (10)	4.2% (15)
Narcotics		
No exposure	88.8% (143)	89.7% (323)
Exposure 1–79%	10.0% (16)	7.5% (27)
Exposure ≥ 80%	1.2% (2)	2.8% (10)
Glucosamine/chondroitin sulfate		
No exposure	45.3% (73)	45.8% (165)
Exposure 1–79%	23.0% (37)	23.1% (83)
Exposure ≥ 80%	31.7% (51)	31.1% (112)

Data shown are proportion of patients (%) and number of patients (*n*)

*COX-2* cyclooxygenase-2, *NSAIDs* non-steroidal anti-inflammatory drugs, *OA* osteoarthritis

^a^Of the total number of cases (218) and controls (540), 161 cases and 360 controls had complete information for the 3 years prior to index date

### Occurrence of KR

The risk of KR occurrence (Table 4) was significantly greater in participants who were white/Caucasian, had a BMI of 27 kg/m^2^ or higher, and had higher WOMAC scores (function, stiffness, total). The KOOS score (pain, symptoms, QoL) was significantly associated with KR occurrence.

**Table 4:** Association between sociodemographic/clinical characteristics^a^ at the index date and occurrence of knee replacement

	Crude OR	95% CI	*p* ^b^
Race: white or Caucasian (reference other race)	1.84	(1.13–2.99)	**0.015**
Education level: college graduate or above (reference less than college graduate)	0.95	(0.65–1.38)	0.778
BMI ≥ 27 kg/m^2^ (reference < 27 kg/m^2^)	1.65	(1.06–2.58)	**0.027**
WOMAC			
Function	1.04	(1.02–1.06)	< **0.001**
Stiffness	1.25	(1.12–1.40)	**0.001**
Total	1.03	(1.02–1.04)	**< 0.001**
KOOS			
Pain	0.98	(0.97–0.99)	< **0.001**
Symptoms	0.98	(0.97–0.99)	< **0.001**
QoL	0.97	(0.96–0.98)	< **0.001**
X-ray (JSW)	0.93	(0.85–1.01)	0.092
MRI			
BML			
By increase of 1%	1.04	(0.99–1.09)	0.144
BML ≥ 1% (reference < 1%)	1.41	(0.95–2.11)	0.091
Meniscal extrusion (reference no extrusion)	1.18	(0.80–1.73)	0.404

*BMI* body mass index, *BML* bone marrow lesion, *CI* confidence interval, *JSW* joint space width, *KOOS* Knee injury and Osteoarthritis Outcome Score, *OR* odds ratio, *QoL* quality of life, *WOMAC* Western Ontario and McMaster Universities Osteoarthritis Index

^a^Excluding characteristics used in the matching between cases and controls (i.e. age, gender, income, WOMAC pain, and KL grade)

^b^Crude conditional logistic regression. Bold indicates statistical significance (*p* < 0.05)

Table 5 presents the adjusted OR of KR occurrence for both primary and secondary analyses according to each class of the oral OA therapies. In the primary analysis (exposure measured in the 3 years prior to index date), none of the oral OA therapeutic classes was significantly associated with the occurrence of KR. Secondary analyses (Table 5) were performed to evaluate the impact of varying time windows of exposure on the occurrence of KR in the adjusted regression analyses. KR occurrence was not associated with exposure to any of the oral OA therapies.

**Table 5:** Association between exposure to different oral osteoarthritis therapies and occurrence of knee replacement

	Acetaminophen				NSAIDs				COX-2 inhibitors				Narcotics				Glucosamine/chondroitin sulfate			
	*n*	OR^b^	95% CI	*p* ^c^	*n*	OR^b^	95% CI	*p* ^c^	*n*	OR^b^	95% CI	*p* ^c^	*n*	OR^b^	95% CI	*p* ^c^	*n*	OR^b^	95% CI	*p* ^c^
Primary analysis																				
In the 3 years prior to index date (*n* = 521: 161 KR/360 controls)																				
Exposure 1–79%^a^	124	1.09	(0.66–1.78)	0.745	313	1.05	(0.64–1.72)	0.839	68	1.13	(0.48–2.67)	0.784	67	1.00	(0.48–2.09)	0.995	120	0.85	(0.50–1.48)	0.573
Exposure ≥ 80%^a^	14	1.11	(0.30–4.09)	0.872	44	1.66	(0.93–2.95)	0.086	21	1.15	(0.47–2.83)	0.761			ND		163	0.73	(0.43–1.23)	0.236
Secondary Analyses																				
In the 2 years prior to index date (*n* = 642: 193 KR/449 controls)																				
Exposure 1–79%^a^	210	1.10	(0.66–1.84)	0.722	369	0.96	(0.60–1.54)	0.853	88	1.40	(0.57–3.48)	0.465	77	1.19	(0.54–2.58)	0.670	255	0.70	(0.39–1.26)	0.235
Exposure ≥ 80%^a^	20	1.46	(0.69–3.11)	0.322	58	1.41	(0.88–2.26)	0.159	27	1.18	(0.55–2.52)	0.671			ND		166	1.00	(0.65–1.55)	0.989
In the 4 years prior to index date (*n* = 363: 121 KR/242 controls)																				
Exposure 1–79%^a^	113	1.34	(0.74–2.41)	0.333	234	1.40	(0.78–2.52)	0.256	50	1.63	(0.64–4.13)	0.303	50	1.43	(0.62–3.33)	0.405	156	0.65	(0.33–1.25)	0.191
Exposure ≥ 80%^a^			ND		26	1.65	(0.75–3.66)	0.215			ND				ND		83	0.63	(0.32–1.21)	0.165
In the 5 years prior to index date (*n* = 228: 82 KR/146 controls)																				
Exposure 1–79%^a^	60	1.38	(0.66–2.88)	0.397	110	1.88	(0.85–4.20)	0.121	18	1.35	(0.41–4.49)	0.623	33	1.19	(0.48–2.93)	0.706	54	0.90	(0.37–2.14)	0.803
Exposure ≥ 80%^a^	15	1.66	(0.44–6.35)	0.457	45	2.27	(0.90–5.71)	0.081			ND				ND		70	0.73	(0.33–1.64)	0.445

*CI* confidence interval, *COX-2* cyclooxygenase-2, *KR* knee replacement, *ND* not determinable, *n* value too small, *NSAIDs* non-steroidal anti-inflammatory drugs, *OA* osteoarthritis, *OR* odds ratio

^a^Compared to the “no exposure” category

^b^Adjusted for race, body mass index, other medication for pain, joint space width, Western Ontario and McMaster Universities Osteoarthritis Index (WOMAC) stiffness, meniscal extrusion, bone marrow lesions, and Knee injury and Osteoarthritis Outcome Score (KOOS)

^c^Conditional logistic regression

## Discussion

This study demonstrated, using the OAI database and a nested case-control study design, that exposure to some of the most commonly used oral OA therapies, i.e. acetaminophen, NSAIDs, COX-2 inhibitors, narcotics, or glucosamine/chondroitin sulfate, in a range of 2– 5 years, was not associated with the occurrence of KR when compared to no exposure to such medications. However, a number of risk factors were identified as being linked to KR, including race, level of symptoms, and BMI.

Our study also revealed that the oral medications studied had a “neutral” effect on KR while controlling for the most important confounding factors known to promote such occurrences: demographics, socioeconomic status, symptom severity, radiographic grading, and structural changes assessed by quantitative MRI. These results are in contrast to those of Hafezi-Nejad et al. [11], also using the same OAI cohort, showing that long-term use of analgesics comprising NSAIDs, acetaminophen, and narcotics alone or in combination may be associated with radiographic progression of knee OA and increased risk of KR. Similar results on the potential deleterious effects of NSAIDs on the evolution of OA structural changes and disease outcome have already been suggested. Early studies based on the radiographic evaluation of disease progression (joint space narrowing (JSN)) in patients with OA treated with NSAIDs reported a negative impact of long-term use of diclofenac or indomethacin in hip and knee OA [4–8]. Another report on the effects of regular use of prescription NSAID treatment in patients with knee OA identified a reduction in JSN compared to non-users over a 4-year follow-up period [10], although the difference between groups was not statistically significant. However, recent observational studies and randomized controlled trials in patients with knee OA using MRI technology to assess disease progression have shown that treatment with NSAIDs such as naproxen or celecoxib (a cyclooxygenase-2 (COX-2) selective inhibitor) has a neutral effect on cartilage loss [26, 27]. Studies from our group, also using MRI technology, and the participants from the incidence and progression subcohorts of the Osteoarthritis Initiative (OAI) database have also explored the effects of NSAIDs/analgesics and glucosamine/chondroitin sulfate on disease progression by assessing the change in cartilage volume [12, 13]. The findings of these studies showed that the extent of progression of cartilage volume loss was driven by disease severity and meniscal extrusion. NSAID/analgesic treatment had no significant effect on cartilage volume loss. In the latter study [13], glucosamine/chondroitin sulfate treatment reduced the cartilage volume loss in participants with meniscal extrusion regardless of whether or not they were receiving NSAID/analgesic treatment.

Interestingly, the findings of Hafezi-Nejad et al. [11] were also not confirmed in the study of Lapane et al. [10] using the same OAI cohort and exploring the effects of long-term use of NSAIDs on knee OA progression also using x-rays. The greater loss of JSW in the NSAID users compared to the non-users was not statistically significant on multivariate adjusted analysis. In another study using the OAI cohort, assessment of disease progression using x-rays and MRI did not demonstrate any effects of long-term use of NSAIDs/analgesics on knee OA disease progression [12, 13]. Finally, in another population-based study, Klop et al. [28] also showed that long-term users of non-selective NSAIDs and coxibs did not have a different risk of KR.

The sociodemographic and clinical data from our study population, being quite similar to those from previous studies exploring the role of disease treatment on KR [8, 10, 11, 28, 29], do not explain the discrepancy in the impact of such NSAIDs. Moreover, studies in preclinical animal models of OA have provided a number of positive as well as negative findings with regard to the potential beneficial or deleterious effects of such drugs on OA structural changes [7, 9], which does not help to settle this debate.

A very important distinction of the present study compared to most others, is the use of a nested case-control design that we chose for a number of reasons. The well-recognized advantage of such a study design is that it allowed us to assess patients’ characteristics as risk factors to be evaluated at the very date of KR surgery. This is in sharp contrast to a cohort study design, in which the patient profiles are assessed at entry (baseline) into the cohort [10, 11, 30]. During the elapsed time between cohort entry and date of KR, which may be several years in most cases, the profiles of patients who undergo KR, such as symptoms, function, and medication usage, may change substantially. The nested case-control design thus allowed evaluation of patient characteristics that best represent the patient status at the time of the KR occurrence, not several years before. It is therefore important to recognize that such a study design will probably impact findings when assessing a relationship between drug exposure and risk of KR, in turn potentially explaining results different from those of recent longitudinal cohort study designs using the same OAI database [11], as mentioned previously.

Another important issue of our study is the use of KR as the sole marker of disease progression, which has already been established as a valid outcome in a number of studies [11, 15–18, 30, 31]. Indeed, there is a general consensus that MRI parameters assessed in knee OA, such as the medial compartment cartilage volume/thickness, can predict outcomes such as KR in a consistent manner [14–18]. However, findings of the present and previous studies [17, 29, 32] also indicate that the ultimate decision of the patient to undergo KR is likely multifactorial in origin and involves a large number of confounding factors that extend well beyond the severity of knee OA structural changes. In our study, however, the cartilage volume at index (KR) time as assessed by MRI was similar in both groups, suggesting that over time the factors leading to progression of cartilage volume loss up to the KR were globally balanced in both groups. One must be cautious with the interpretation that drug treatment that can accelerate disease progression, if true, may have exerted its effects on both control and KR groups. Although the effect of drug treatment on rate of disease progression was not assessed in this study, several previous studies have addressed this very specific issue, a number of which used the OAI cohort [10–13]. Based on the findings of these studies, one may be tempted to conclude that factors in addition to disease progression are very likely to influence the patient’s decision to undergo KR. The results of the present study showing that JSW at index time was not linked to the occurrence of KR certainly support this view.

In the present study we also explored the cumulative exposure to oral OA therapies measured in different time windows, from 2 to 5 years before the date of the KR, to evaluate their impact in different scenarios to yield robustness of our analyses. Interestingly, these analyses did not show any time frame trends, shorter or longer, that would significantly promote greater risk of KR. Windows of 6 to 8 years of exposure to oral OA therapies were also considered, but the number of available patients was too small for statistical inference.

The results of the study are reassuring and clinically relevant since they tackle the confounding role of any oral intervention to treat pain as a “last resort” prior to inevitable surgery, creating a spurious association between drug usage and the risk of KR, in turn suggesting a deleterious role of the medication via a channeling bias. Moreover, presence of severe comorbidities, frequently encountered with severe OA, are usually perceived by orthopedic surgeons as promoting perioperative risks and, as such, they are less inclined to recommend surgery for these patients. These same comorbidities may also preclude the use of NSAIDs and narcotics for these “morbid” patients, hence yielding spurious correlation between use of these medications and more KRs being performed.

Our study has a number of strengths. First, it was conducted using the large OAI database, representative of a North American population with fairly open access to usual care for knee OA, including KR, based on patient and physician preferences in a context of a real-world scenario. Second, to our knowledge, this is among the first studies that allowed stratification of risk of KR by extent of exposure to oral OA medication versus no exposure, which is paramount when trying to establish a cause-effect of medication on an ultimate outcome such as KR. We chose a priori a 3-year window of oral OA medication exposure based on a clinical rationale and the design of previous studies [17, 30, 31]. Third, per the OAI design, we have great certainty about the knee OA diagnosis and its KR indication based on detailed information on demographics, symptoms, imaging, and drug use for both patients and their matched controls. Fourth, medical data were routinely recorded by investigators including rheumatologists and orthopedic surgeons without a study hypothesis, yielding a “nested” case-control study, hence minimizing the possibility of recall bias, which plagues conventional retrospective chart review studies. Last, the excellent matching yielded from our control selection strategy, as shown in the baseline characteristics comparison, is reassuring, as control selection is always an important issue for nested case-control designs as poor choices may yield very different conclusions.

Limitations of this study include the fact that it did not allow identification of any specific drug class. For instance, the impossibility of defining a specific NSAID name within the database is a limitation as some NSAIDs, such as for example indomethacin, may prove to be more deleterious than others [4–9]. The data provided on drug usage were obtained by self-administered questionnaire and not by a traditional pill count, which is used to assess adherence and persistence to medication in most clinical trials. This could underestimate true prolonged and cumulative usage of these medications. We have nonetheless tried to establish a “dose-effect” response using categories we have coined levels of “exposure”: no exposure, occasional (exposure 1–79%), or regular (≥ 80%) medication usage, acknowledging such limitation.

The study design also did not allow comprehensive assessment of the influence of confounding factors, as some of the data used were only available at baseline and not at the index time of KR. Despite attempts to adjust for several confounders, causal interpretation of the findings is restricted, and residual confounding must be considered when interpreting the results.

Furthermore, this study focused on patients with severe OA in need of surgery; other beneficial or deleterious associations with chronic use of oral OA drugs and subclinical structural damage, as seen using quantitative MRI for example, may be found in subjects with less severe OA.

It was also impossible to assess the knee OA disease duration since onset of symptoms or date of OA diagnosis was not collected in the OAI dataset. Knee OA duration could have a significant impact on the cumulative and progressive joint damage, but we were unfortunately unable to control for it.

Statistical power may also be an issue since, by selecting subjects that had a KR but also had almost all demographic, clinical and MRI information, patient number was reduced from more than 4674 subjects to a mere 218 cases of KR, which is somewhat limited for performing multivariate analyses.

Finally, actual KR occurrences may be considered by some as an inadequate outcome for a comprehensive severe progressive OA definition. In fact, in the present work, we did not assess knee OA progression using imaging outcomes such as JSW or cartilage thickness/volume loss prior to the KR. The rate of such progression might accelerate while nearing the KR occurrence, which may or may not be associated with oral OA medication use. However, as already mentioned, the cartilage volume at index (KR) time as assessed by MRI was similar in both groups, suggesting that over time the factors leading to progression of cartilage volume loss up to the KR were globally balanced in both groups. Despite the great clinical success of KR, the criteria on which surgery is performed are not uniform. Apart from symptoms and radiographic status, surgical indication depends on willingness, comorbidity, access to health care, socioeconomic status, etc. A validated KR “indication” as a clinical outcome, as suggested by the OARSI/Outcome Measures in Rheumatology (OMERACT) group [32] could help in that regard for future studies.

## Conclusion

The present study indicates that patients chronically taking the most commonly used oral OA therapies do not have an increased risk of KR. In an era of OA therapeutic choice paucity, our study is somewhat reassuring and repositions chronic symptomatic OA treatment as safe. However, longer-term and controlled studies and safety assessments should also be performed in the context of longitudinal follow up to further probe our initial findings.

## Abbreviations

BMI: Body mass index
BML: Bone marrow lesion
CI: Confidence interval
COX-2: Cyclooxygenase-2
IQR: Interquartile range
JSW: Joint space width
KL: Kellgren-Lawrence
KOOS: Knee injury and osteoarthritis outcome score
KR: Knee replacement
MRI: Magnetic resonance image
NSAIDs: Non-steroidal anti-inflammatory drugs
OA: Osteoarthritis
OAI: Osteoarthritis Initiative
OARSI: Osteoarthritis Research Society International
OR: Odds ratio
QoL: Quality of life score
WOMAC: Western Ontario and McMaster Universities Osteoarthritis Index

## References

[1] Hagen KB, Smedslund G, Moe RH, Grotle M, Kjeken I, Kvien TK (2009). The evidence for non-pharmacological therapy of hand and hip OA. Nat Rev Rheumatol..

[2] FDA Drug Safety Communication. FDA strengthens warning that non-aspirin nonsteroidal anti-inflammatory drugs (NSAIDs) can cause heart attacks or strokes (7-9-2015). Available at: https://www.fda.gov/Drugs/DrugSafety/ucm451800.htm (Accessed 23 May 2018).

[3] Solomon DH, Rassen JA, Glynn RJ, Lee J, Levin R, Schneeweiss S (2010). The comparative safety of analgesics in older adults with arthritis. Arch Intern Med..

[4] Milner JC (1972). Osteoarthritis of the hip and indomethacin. J Bone Joint Surg (Br)..

[5] Ronningen H (1977). Indomethacin hips. Acta Orthop Scand..

[6] Huskisson EC, Berry H, Gishen P, Jubb RW, Whitehead J (1995). Effects of antiinflammatory drugs on the progression of osteoarthritis of the knee. LINK study group. Longitudinal investigation of nonsteroidal antiinflammatory drugs in knee osteoarthritis. J Rheumatol..

[7] Ding C (2002). Do NSAIDs affect the progression of osteoarthritis?. Inflammation..

[8] Reijman M, Bierma-Zeinstra SM, Pols HA, Koes BW, Stricker BH, Hazes JM (2005). Is there an association between the use of different types of nonsteroidal antiinflammatory drugs and radiologic progression of osteoarthritis? The Rotterdam study. Arthritis Rheum..

[9] Hauser RA (2010). The acceleration of articular cartilage degeneration in osteoarthritis by nonsteroidal anti-inflammatory drugs. J Prolotherapy..

[10] Lapane KL, Yang S, Driban JB, Liu SH, Dube CE, McAlindon TE, Eaton CB (2015). Effects of prescription nonsteroidal antiinflammatory drugs on symptoms and disease progression among patients with knee osteoarthritis. Arthritis Rheumatol..

[11] Hafezi-Nejad N, Guermazi A, Roemer FW, Eng J, Zikria B, Demehri S (2016). Long term use of analgesics and risk of osteoarthritis progressions and knee replacement: propensity score matched cohort analysis of data from the osteoarthritis initiative. Osteoarthr Cartil..

[12] Martel-Pelletier J, Roubille C, Abram F, Hochberg MC, Dorais M, Delorme P, Raynauld JP, Pelletier JP (2015). First-line analysis of the effects of treatment on progression of structural changes in knee osteoarthritis over 24 months: data from the osteoarthritis initiative progression cohort. Ann Rheum Dis..

[13] Roubille C, Martel-Pelletier J, Abram F, Dorais M, Delorme P, Raynauld JP, Pelletier JP (2015). Impact of disease treatments on the progression of knee osteoarthritis structural changes related to meniscal extrusion: data from the OAI progression cohort. Semin Arthritis Rheum..

[14] Eckstein F, Boudreau RM, Wang Z, Hannon MJ, Wirth W, Cotofana S, Guermazi A, Roemer F, Nevitt M, John MR (2014). Trajectory of cartilage loss within 4 years of knee replacement–a nested case-control study from the osteoarthritis initiative. Osteoarthr Cartil..

[15] Eckstein F, Kwoh CK, Boudreau RM, Wang Z, Hannon MJ, Cotofana S, Hudelmaier MI, Wirth W, Guermazi A, Nevitt MC (2013). Quantitative MRI measures of cartilage predict knee replacement: a case-control study from the osteoarthritis initiative. Ann Rheum Dis.

[16] Pelletier JP, Cooper C, Peterfy C, Reginster JY, Brandi ML, Bruyere O, Chapurlat R, Cicuttini F, Conaghan PG, Doherty M (2013). What is the predictive value of MRI for the occurrence of knee replacement surgery in knee osteoarthritis?. Ann Rheum Dis..

[17] Raynauld JP, Martel-Pelletier J, Haraoui B, Choquette D, Dorais M, Wildi LM, Abram F, Pelletier JP (2011). Risk factors predictive of joint replacement in a 2-year multicentre clinical trial in knee osteoarthritis using MRI: results from over 6 years of observation. Ann Rheum Dis..

[18] Cicuttini FM, Jones G, Forbes A, Wluka AE (2004). Rate of cartilage loss at two years predicts subsequent total knee arthroplasty: a prospective study. Ann Rheum Dis..

[19] Breslow NE, Day NE, Breslow NE, Day NE (1987). Chapter 7. Design considerations. Table 7.9. Statistical methods in cancer research. Volume II–the design and analysis of cohort studies.

[20] Bellamy N, Buchanan WW, Goldsmith CH, Campbell J, Stitt LW (1988). Validation study of WOMAC: a health status instrument for measuring clinically important patient relevant outcomes to antirheumatic drug therapy in patients with osteoarthritis of the hip or knee. J Rheumatol..

[21] Roos EM, Roos HP, Lohmander LS, Ekdahl C, Beynnon BD (1998). Knee injury and osteoarthritis outcome score (KOOS)–development of a self-administered outcome measure. J Orthop Sports Phys Ther..

[22] Dodin P, Pelletier JP, Martel-Pelletier J, Abram F (2010). Automatic human knee cartilage segmentation from 3D magnetic resonance images. IEEE Trans Biomed Eng..

[23] Dodin P, Abram F, Pelletier J-P, Martel-Pelletier J (2013). A fully automated system for quantification of knee bone marrow lesions using MRI and the osteoarthritis initiative cohort. J Biomed Graph Comput..

[24] Berthiaume MJ, Raynauld JP, Martel-Pelletier J, Labonté F, Beaudoin G, Bloch DA, Choquette D, Haraoui B, Altman RD, Hochberg M (2005). Meniscal tear and extrusion are strongly associated with the progression of knee osteoarthritis as assessed by quantitative magnetic resonance imaging. Ann Rheum Dis..

[25] Raynauld JP, Martel-Pelletier J, Berthiaume MJ, Beaudoin G, Choquette D, Haraoui B, Tannenbaum H, Meyer JM, Beary JF, Cline GA (2006). Long term evaluation of disease progression through the quantitative magnetic resonance imaging of symptomatic knee osteoarthritis patients: correlation with clinical symptoms and radiographic changes. Arthritis Res Ther..

[26] Raynauld JP, Martel-Pelletier J, Beaulieu A, Bessette L, Morin F, Choquette D, Haraoui B, Abram F, Pelletier JP (2010). An open-label pilot study evaluating by magnetic resonance imaging the potential for a disease-modifying effect of celecoxib compared to a modelized historical control cohort in the treatment of knee osteoarthritis. Semin Arthritis Rheum..

[27] Raynauld JP, Martel-Pelletier J, Bias P, Laufer S, Haraoui B, Choquette D, Beaulieu AD, Abram F, Dorais M, Vignon E (2009). Protective effects of licofelone, a 5-lipoxygenase and cyclo-oxygenase inhibitor, versus naproxen on cartilage loss in knee osteoarthritis: a first multicentre clinical trial using quantitative MRI. Ann Rheum Dis..

[28] Klop C, de Vries F, Lalmohamed A, Mastbergen SC, Leufkens HG, Noort-van der Laan WH, Bijlsma JW, Welsing PM (2012). COX-2-selective NSAIDs and risk of hip or knee replacements: a population-based case-control study. Calcif Tissue Int..

[29] Riddle DL, Kong X, Jiranek WA (2009). Two-year incidence and predictors of future knee arthroplasty in persons with symptomatic knee osteoarthritis: preliminary analysis of longitudinal data from the osteoarthritis initiative. Knee..

[30] Bruyere O, Pavelka K, Rovati LC, Gatterova J, Giacovelli G, Olejarova M, Deroisy R, Reginster JY (2008). Total joint replacement after glucosamine sulphate treatment in knee osteoarthritis: results of a mean 8-year observation of patients from two previous 3-year, randomised, placebo-controlled trials. Osteoarthr Cartil..

[31] Raynauld JP, Pelletier JP, Abram F, Dodin P, Delorme P, Martel-Pelletier J (2016). Long-term effects of glucosamine and chondroitin sulfate on the progression of structural changes in knee osteoarthritis: six-year follow up data from the osteoarthritis initiative. Arthritis Care Res (Hoboken)..

[32] Gossec L, Paternotte S, Bingham CO, Clegg DO, Coste P, Conaghan PG, Davis AM, Giacovelli G, Gunther KP, Hawker G (2011). OARSI/OMERACT initiative to define states of severity and indication for joint replacement in hip and knee osteoarthritis. An OMERACT 10 special interest group. J Rheumatol..

